# cgmisc: enhanced genome-wide association analyses and visualization

**DOI:** 10.1093/bioinformatics/btv426

**Published:** 2015-08-06

**Authors:** Marcin Kierczak, Jagoda Jabłońska, Simon K. G. Forsberg, Matteo Bianchi, Katarina Tengvall, Mats Pettersson, Veronika Scholz, Jennifer R. S. Meadows, Patric Jern, Örjan Carlborg, Kerstin Lindblad-Toh

**Affiliations:** 1 ^1^Science for Life Laboratory, Department of Medical Biochemistry and Microbiology, Uppsala University, Uppsala, Sweden,; 2 ^2^Computational Genetics Section, Department of Clinical Sciences, Swedish University of Agricultural Sciences, Uppsala, Sweden and; 3 ^3^Broad Institute of MIT and Harvard, Boston, MA, USA

## Abstract

**Summary:** High-throughput genotyping and sequencing technologies facilitate studies of complex genetic traits and provide new research opportunities. The increasing popularity of genome-wide association studies (GWAS) leads to the discovery of new associated loci and a better understanding of the genetic architecture underlying not only diseases, but also other monogenic and complex phenotypes. Several softwares are available for performing GWAS analyses, R environment being one of them.

**Results:** We present cgmisc, an R package that enables enhanced data analysis and visualization of results from GWAS. The package contains several utilities and modules that complement and enhance the functionality of the existing software. It also provides several tools for advanced visualization of genomic data and utilizes the power of the R language to aid in preparation of publication-quality figures. Some of the package functions are specific for the domestic dog (*Canis familiaris*) data.

**Availability and implementation:** The package is operating system-independent and is available from: https://github.com/cgmisc-team/cgmisc

**Contact:** marcin.kierczak@imbim.uu.se

**Supplementary information:** Supplementary data are available at *Bioinformatics* online.

## 1 Introduction

**Fig. 1.**
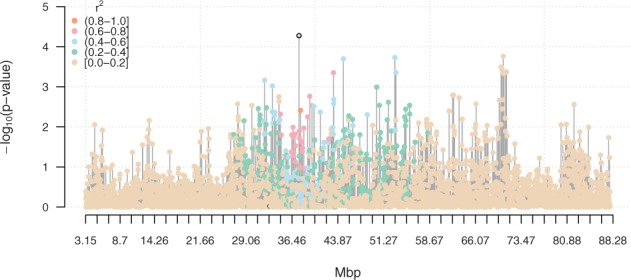
An example figure generated using the cgmisc package: p-values from Fisher’s exact test for allele counts highlight the most divergent regions between two populations. Colour of the points correspond to their LD (*r*^2^) with the most significant marker (the reference)

High-throughput genotyping and sequencing has opened several new research opportunities to study complex genetic traits and genome-wide association studies (GWAS) is a popular way to analyse genotyping data from segregating populations. Widely used GWAS softwares include PLINK (Purcell *et* *al.*, 2007), EMMAX (Kang *et* *al.*, 2010), GCTA (Yang *et* *al.*, 2011) and GenABEL (Aulchenko *et* *al.*, 2007). A single software package is rarely sufficiently complete to cover all aspects of a typical genome-wide analysis pipeline. Transferring data between different softwares is often a laborious process. One advantage of the GenABEL package, that often makes it the software of choice, is that in addition to GWAS-specific functionalities, it provides access to the R (R Development Core Team, 2008) language and community-contributed packages. Here we developed a number of algorithms and solutions to several common GWAS tasks. Some of these solutions aim at facilitating production of publication-quality data/results visualization (see, e.g. Fig. 1). Several cgmisc functions have been used to produce results and visualizations for peer-reviewed publications, e.g. Tengvall *et* *al.*, 2013, Owczarek-Lipska *et* *al.*, 2012 and Olsson *et* *al.*, 2013. Here, we present these and more functions in the form of the documented and supported R package cgmisc.

## 2 Description

cgmisc (ver. 2.9.10), provides 34 functions for the analysis and visualization of GWAS data. A few functions in the package are tailored for working with data from the domestic dog (*Canis familiaris*) but are easy to adjust for analysing other species. Some functions rely on third-party softwares which are freely available for research purposes. Internally, cgmisc functions use data structures implemented in the GenABEL package. For all functions and parameters in the package, we use the period-separated naming convention (Bååth, 2012). Functions provided by the cgmisc package can be grouped into the following categories:

- **Analyses of population structure.** Population strata can be compared based on their allele-frequency differences, using either (i) fixation index *F_ST_* or (ii) Fisher’s exact test for reference allele count observed versus the allele count expected under the null hypothesis of no population structure.
- **Tools related to association scans.** Enhanced quantile–quantile (qq) plot showing (i) theoretical and (ii) empirical confidence intervals as well as (iii) empirical significance thresholds. We also implemented an extended version of the Manhattan plot, with colour-coded information on linkage disequilibrium (LD) between a selected marker and its neighbours plus a minor-allele frequency panel. Easy ways of interfacing variance GWAS scans (vGWAS; Shen *et* *al.*, 2012) and bigRR (Shen *et* *al.*, 2013) packages (BLUP, ridge regression) as well as simple visualization of per-genotype distribution of phenotypic values are provided. We also complement the standard tests for association with a basic scan for gene-gene interaction (epistasis).
- **Heterozygosity analyses.** We provide functions for the detection and visualization of runs of homozygosity along the genome to facilitate the detection of suggestive selective sweeps and highlight regions that may be challenging for standard association mapping tools.
- **Analyses and visualization of linkage structure.** The cgmisc package provides tools for assessing average haplotype lengths by visualization of LD-decay as a function of the distance between markers. In addition, the package offers export functions that enable haplotype phasing using PHASE (Stephens *et* *al.*, 2001) and haplotype visualization using Haploview (Barrett *et* *al.*, 2005). In addition, we implemented the marker clumping procedure used by PLINK.
- **Improved annotation.** The package provides functions for genome annotation in the domestic dog (*Canis familiaris*, canFam3.1 assembly), offers the direct interaction with the UCSC Genome Browser (Kuhn *et* *al.*, 2013) and improved analyses of pseudo-autosomal regions on the X chromosome. In addition, we provide a convenient method for retrieving and plotting information on endogenous retroviral sequences identified by the RetroTector software (Sperber *et* *al.*, 2007).
- **Data subsetting, manipulation and visualization.** cgmisc can generate windows for sliding-window (also with overlap) and jumping-window type analyses. A number of convenience functions enables users to, e.g. retrieve information about LD or chromosome start/end point coordinates.

All functions were designed to be user-friendly with attention to the quality of visualizations. Complete documentation is available upon cgmisc installation. In order to facilitate package usage, we included a quick tutorial (package vignette in the supplementary information) that takes the user through all steps necessary to use each of the package functions. The tutorial is based on the included example dataset. A detailed description of the methods and algorithms used by the functions is provided in the vignette and documentation.

## Funding

M.K. was supported by the Swedish Foundation for Strategic Research. J.R.S.M., M.K. and Ö.C. received support from FORMAS. M.K., S.F. and Ö.C. were supported by the Swedish Research Council. K.L.-T. and M.K. were supported by the European Research Council. J.J. was supported by the European Commission, Erasmus mobility grant.

*Conflict of Interest*: none declared.

## Supplementary Material

Supplementary Data
