# Supplementary material for: cgmisc: enhanced genome-wide association analyses and visualization
Source: Bioinformatics. 2015 Aug 6;31(23):3830–1. doi: 10.1093/bioinformatics/btv426 (PMC4653382; doi:10.1093/bioinformatics/btv426)
Supplement: Supplementary Data [file supp_31_23_3830__index.html]

cgmisc: Enhanced Genome-wide Association Analyses and Visualisation — cgmisc: enhanced genome-wide association analyses and visualization — cgmisc: enhanced genome-wide association analyses and visualization — Supplementary Data 

# cgmisc: enhanced genome-wide association analyses and visualization

## Supplementary Data

files

- Supplementary Data - pdf file
