# Supplementary material for: cgmisc: enhanced genome-wide association analyses and visualization
Source: Bioinformatics. 2015 Aug 6;31(23):3830–1. doi: 10.1093/bioinformatics/btv426 (PMC4653382; doi:10.1093/bioinformatics/btv426)
Supplement: Supplementary Data [file supp_btv426_vignette.pdf]

cgmisc: package tutorial

Marcin Kierczak, Jagoda Jabłońska, Simon Forsberg, Matteo Bianchi, Katarina Tengvall, Mats Pettersson, Veronika Scholz, Jennifer Meadows, Patric Jern, Örjan Carlborg, Kerstin Lindblad-Toh

17 June 2015

Contents

Synopsis **2**

Package installation **2**

Loading data **2**

Example analyses **2**

Quality control . . . 2
Detecting population structure . . . 3
Comparing subpopulations . . . 4
Association analyses . . . 6
Visualising and analysing linkage structure . . . 7
Visualising gene annotations . . . 8
Working with genomic regions . . . 9
Clumping procedure . . . 10
Improved quantile-quantile plots . . . 10
Interacting with the UCSC genome browser . . . 12
Examining LD decay . . . 12
Detecting runs of homozygosity . . . 13
Computing average heterozygosity using overlapping windows approach . . . 13
Examining allele/genotype effect using phenotype boxplots . . . 14
Simple epistasis scan . . . 15
Data conversion functions . . . 16
Dog-specific utilities . . . 17
Endogenous retroviral sequences (ERV) . . . 17

List of function aliases **18**

References **18**

Synopsis

cgmisc is an R package for enhanced data analyses and visualisation in genome-wide association studies (GWAS). This document will guide you through the installation process and to demonstrate package capabilities in a series of practical examples based on a showcase data included in the package.

Package installation

The **cgmisc** package is available on GitHub repository and can be installed with the help of the `devtools` package:

```
install.packages('devtools') # Install devotools if not installed
library(devtools) # Load the library
install_github('cgmisc-team/cgmisc') # Install cgmisc
```

In order to use the package functions, it is necessary to load it into environment:

```
library('cgmisc')
```

Loading data

Whenever possible, the **cgmisc** package works with data structures implemented and used by the GenABEL (Aulchenko et al. 2007) package. In particular, the `gwaa.data-class` and the `gwaa.scan-class` structures are used. The package is shipped with an example dataset called **cgmisc_data**. The example dataset contains genotyping data (Illumina CanineHD array, canFam2 assembly) for N=207 German shepherds originally collected for the project described in (Tengvall et al. 2013). However, to illustrate various features of the **cgmisc** package, the phenotypes included in the example dataset have been simulated. Use the following command to load the example dataset:

```
data('cgmisc_data')
```

Now, once the data have been loaded, we can start analyses.

Example analyses

In order to illustrate how to use particular functions, we will perform a **much** simplified GWAS analysis. We begin by performing initial quality control.

Quality control

First, we will prune the data with *per marker* or *per individual* call rates below 95%. Based on 2000 randomly selected autosomal markers, we remove one (the one with lower call rate) from each pair of too similar (more than 95% similarity) individuals. We also set very low (10^{-3}) threshold for pruning on minor allele frequency (in practise only the monomorphic markers will be removed) and turn off checks based on the departure form Hardy-Weinberg equilibrium (p.level=10e-18)

```r
qc1 <- check.marker(data = data,
                    callrate = .95,
                    perid.call = .95,
                    ibs.threshold = .95,
                    ibs.mrk = 2000,
                    ibs.exclude = "lower",
                    p.level = 10e-18,
                    maf = 1e-3)
data.qc1 <- data[qc1$idok, qc1$snpok]
```

Detecting population structure

Now, we will analyse population structure using genomic-kinship information. Using all autosomal chromosomes, we will calculate the Identity By State (IBS) matrix which captures pairwise genetic similarity between individuals. Then, in order to visualize population structure, we will use the multidimensional scaling (MDS) technique to reduce the IBS matrix to two dimensions.

```r
autosomal <- which(data.qc1@gtdata@chromosome != 39) # 39 is the canine X chromosome
data.qc1.gkin <- ibs(data.qc1, snpsubset = autosomal, weight = 'freq')
data.qc1.dist <- as.dist(0.5 - data.qc1.gkin) # Compute pairwise distances
data.qc1.mds <- cmdscale(data.qc1.dist) # Multidimensional scaling
plot(data.qc1.mds, pch = 19, cex = .5, las = 1,
     xlab = 'MDS1', ylab = 'MDS2',
     cex.axis = .7, bty = 'n')
```

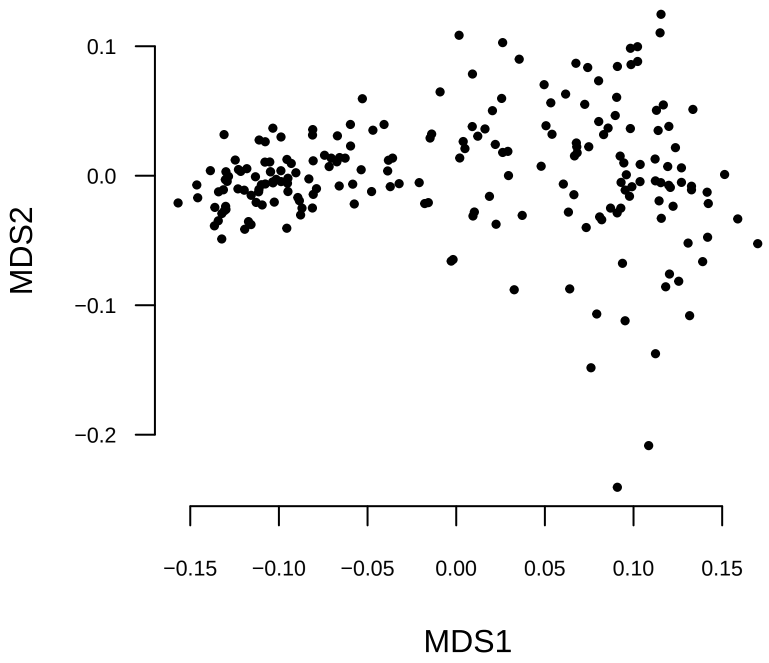

We can see that there is a chance of population structure here: a somewhat tighter cluster of individuals to the left. We should investigate this further, but for the purpose of this tutorial, let's just run simple K-means clustering with the number of clusters *a priori* set to $K = 2$.

```
kclust <- kmeans(data.qc1.mds, centers = 2)
plot(data.qc1.mds, pch = 19, cex = .5, las = 1,
     xlab = 'MDS1', ylab = 'MDS2', cex.axis = .7,
     bty = 'n', col = kclust$cluster)
```

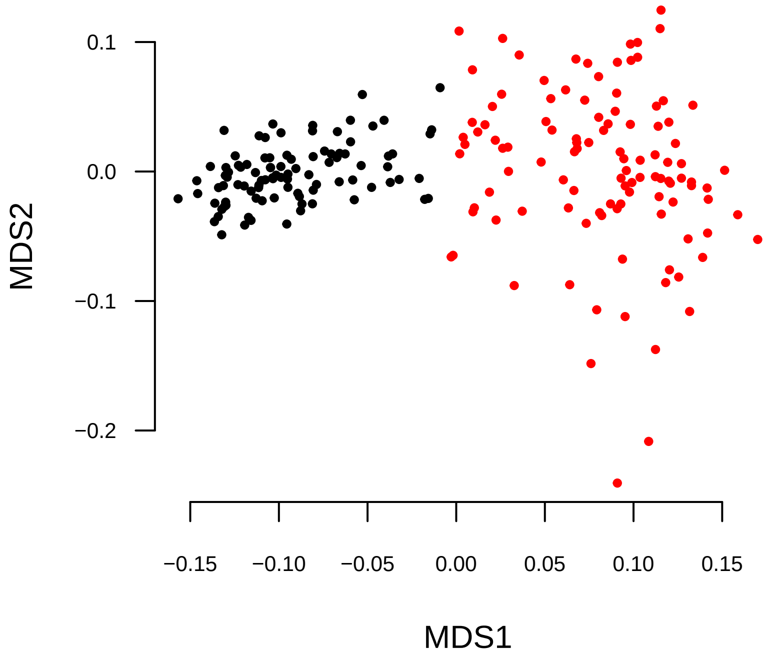


```
# Now, we can assign our individuals to the calculated
# clusters which represent subpopulations.
pop <- kclust$cluster
```

Comparing subpopulations

We can compare subpopulations by, e.g. looking at the differences in the reference allele frequency using the `pop.allele.counts` and the `plot.pac` functions. Here, we will focus on the chromosome 2 only.

Comparing of allele counts

We can perform Fisher's exact test on the observed per-population reference allele counts and the counts expected assuming null hypothesis of no population structure (all individuals drawn from the same population). Significant deviations from this expected frequency mark divergent regions.

```
pac <- pop.allele.counts(data = data.qc1[ ,data.qc1@gtdata@chromosome == 2],
                         pops = pop,
                         progress=F)
plot.pac(data = data.qc1[ ,data.qc1@gtdata@chromosome == 2],
         allele.cnt = pac,
         plot.LD = T,
         legend.pos='topleft')
```

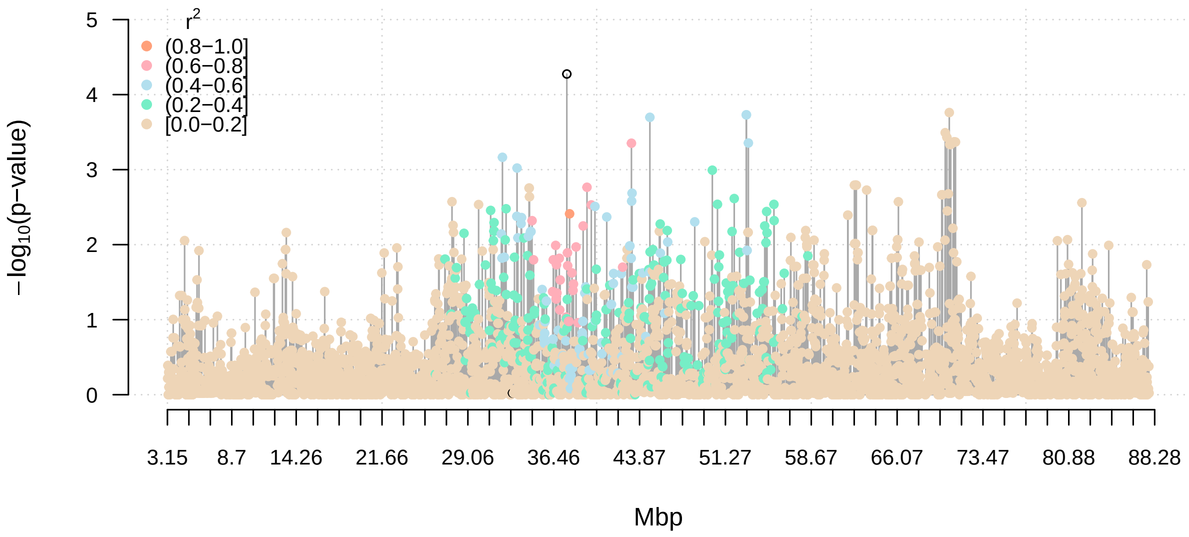

Computing fixation index F_{ST}

In a similar way, we can compute and plot fixation index F_{ST}:

```
fst <- compute.fstats(data = data.qc1[ ,data.qc1@gtdata@chromosome == 2],
                      pops = pop)
plot.fstats(data = data.qc1[ ,data.qc1@gtdata@chromosome == 2],
            fstats = fst,
            est.type = 'naive')
```

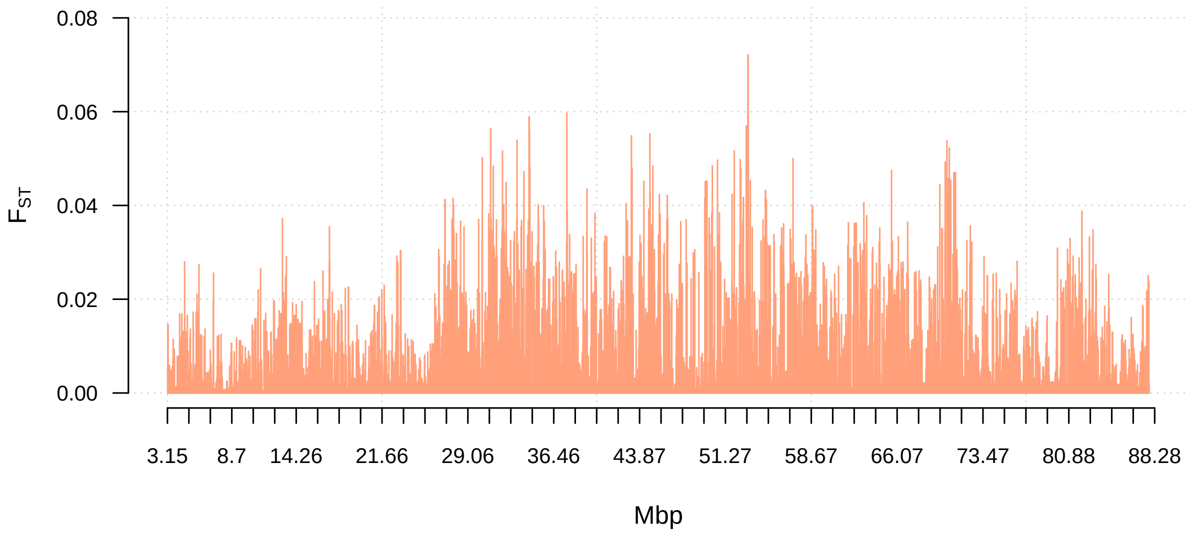

Association analyses

Having defined and briefly analysed the subpopulations, we can proceed to association analyses using, e.g. mixed model with genomic kinship as random effect.

```
h2h <- polygenic(formula = ct ~ sex,
                 kinship.matrix = data.qc1.gkin,
                 data = data.qc1,
                 llfun = 'polylik')
mm <- mmscore(h2object = h2h, data = data.qc1, strata = pop)
```

The per-marker significance of associations can be now plotted (Manhattan plot):

```
par(las = 1, cex.axis = .7) # Tweak graphics
plot(mm, cex = .5, pch = 19, col = c('darkgrey', 'grey'))
```

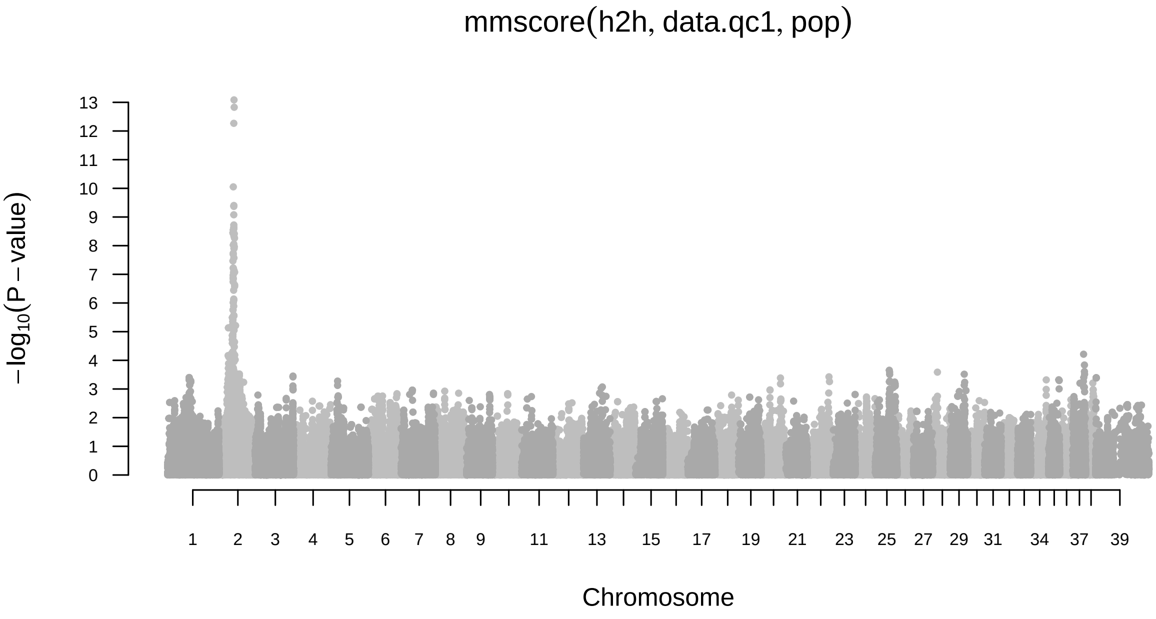

As we can see, there is a very strong association signal on chromosome 2. We can examine it a bit closer using the `plot.manhattan.ld` function.

Visualising and analysing linkage structure

Say, we would like to zoom in on chromosome 2 and visualise linkage disequilibrium (LD) of every marker in the region to the top-associated marker. First, we need the name and coordinates of the top-associated marker:

```
summary(mm, top = 1)
```

```
## Summary for top 1 results, sorted by P1df
```

```
##               Chromosome Position Strand A1 A2   N     effB   se_effB
## BICF2S2365880          2 38256927      u  T  C 205 1.097095 0.1469296
##               chi2.1df         P1df        Pc1df effAB effBB chi2.2df P2df
## BICF2S2365880 55.75325 8.216247e-14 3.997282e-13    NA    NA        0   NA
```

The top-associated marker is **BICF2S2365880** and its position is **38256927bp**. We will zoom in on a 2 Mbp region centered on the marker using the `plot.manhattan.ld` function. The function produces a standard so-called Manhattan plot with color-coded linkage disequilibrium (LD) to a specific reference marker measured by r^2. Given genotyping data (as GenABEL `gwaa.data-class` object) and GWAS result in the form of p-values vector together with genetic coordinates of a region to be plotted (up to entire chromosome), plot.manhattan.ld produces a plot with genomic coordinates on the x-axis and $-log_{10}(p-value)$ on the y-axis. Linkage disequilibrium to a reference marker is represented by specific colors. By default, colors represent 5 discrete intervals of r^2: $[1.0, 0.8[$; $[0.8, 0.6[$; $[0.6, 0.4[$; $[0.4, 0.2[$ and [0.2, 0[, where: $[a, b[:= x : a >= x > b$. If desired, the intervals can be altered using function parameters.

```
plot.manhattan.LD(data = data.qc1,
                  gwas.result = mm,
                  chr = 2,
                  region = c(37256927, 39256927),
                  index.snp = 'BICF2S2365880',
                  legend.pos = 'topleft')
```

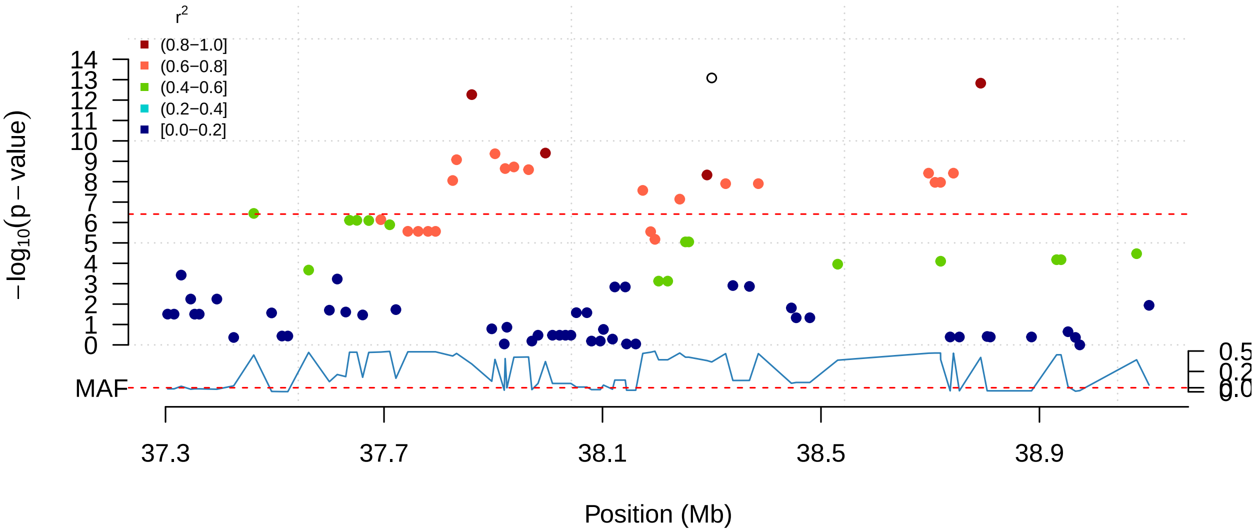

Visualising gene annotations

The `plot.manhattan.genes` extends the `plot.manhattan.ld` function by enabling visualisation of genes provided in a BED file. In the **cgmisc** package, we provide a BED file containing information on protein coding genes in dog canFam3.1 assembly. The file was prepared using Broad Improved Canine Annotation v.1 available at the UCSC Genome Browser. Below, we use this file to visualise genes in a specified region:

```
fpath <- system.file('extdata', 'canFam3.1.prot.bed', package = 'cgmisc')
plot.manhattan.genes(data = data.qc1,
                     gwas.result = mm,
                     chr = 2,
                     region = c(37256927, 39256927),
                     index.snp = 'BICF2S2365880',
                     bed.path=fpath)
```

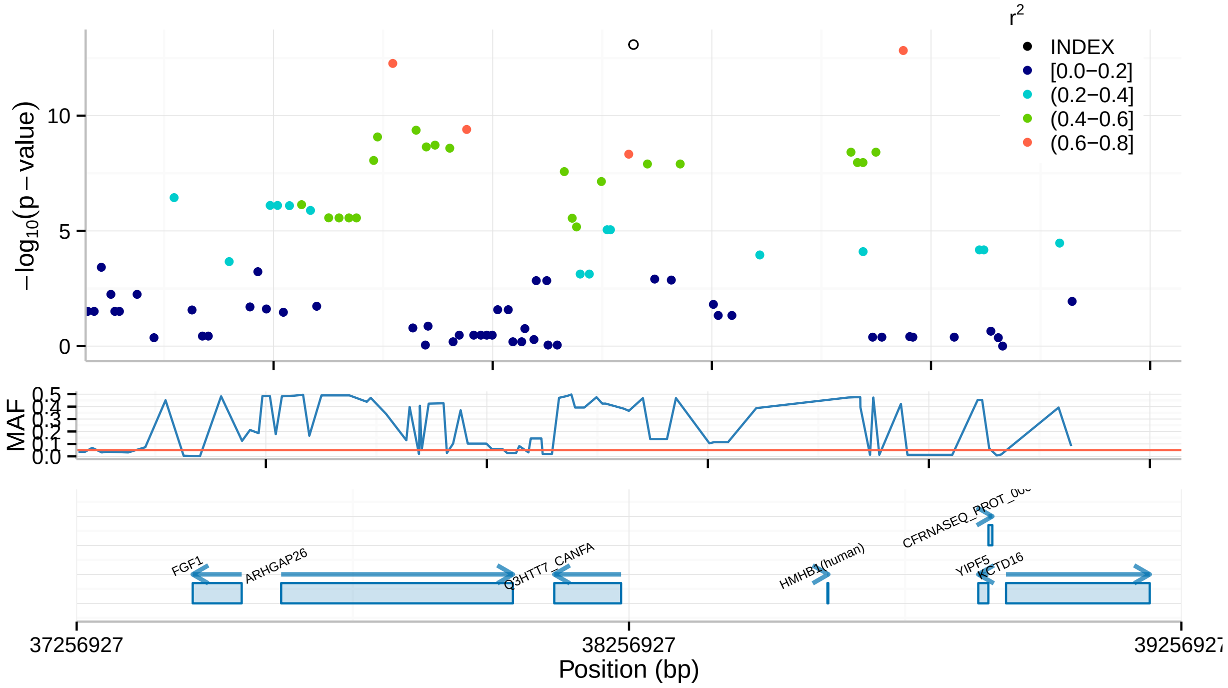

Working with genomic regions

To extract markers from a given genomic region that are in high LD to a given index marker the `choose.top.snps` function can be used.

```
top.snps <- choose.top.snps(data = data.qc1,
                            chr = 2,
                            region = c(37256927, 39256927),
                            index.snp = 'BICF2S2365880')
print (top.snps[1:4,])
```

```
##                                 r2    coord
## BICF2S2365880          INDEX SNP 38256927
## BICF2P462003   0.916279689260684 37817567
## BICF2P425207   0.866030555369713 38248275
## BICF2P612394   0.843123203444902 37952464
```

To extract markers within the user-defined (in bp) neighborhood of a given marker, one can use the `get.adjacent.markers` function.

```
adjacent <- get.adjacent.markers(data = data.qc1,
                                 marker = 'BICF2S2365880',
                                 size.bp = 1e4)
print(adjacent[1:4, ])
```

```
##         BICF2P425207 BICF2S2365880
## dog224             1             1
## dog225             0             0
## dog226             1             1
## dog227             1             1
```

Clumping procedure

We can also use the clumping procedure as outlined in PLINK documentation (http://pngu.mgh.harvard.edu/purcell/plink/clump.shtml) to single out regions of interest. As we can see, there are two clumps represented by grey and violet points respectively. The clumps are shown on both the standard Manhattan plot (upper panel) and, for improved clarity, also on a dedicated clump panel (lower panel). In short: a clump contains markers in high LD that are also significantly associated with the examined trait.

```
clumps <- clump.markers(data = data,
                        gwas.result = mm,
                        chr = 2)
plot.clumps(gwas.result = mm,
            clumps = clumps,
            chr = 2,
            region = c(37256927, 39256927))
```

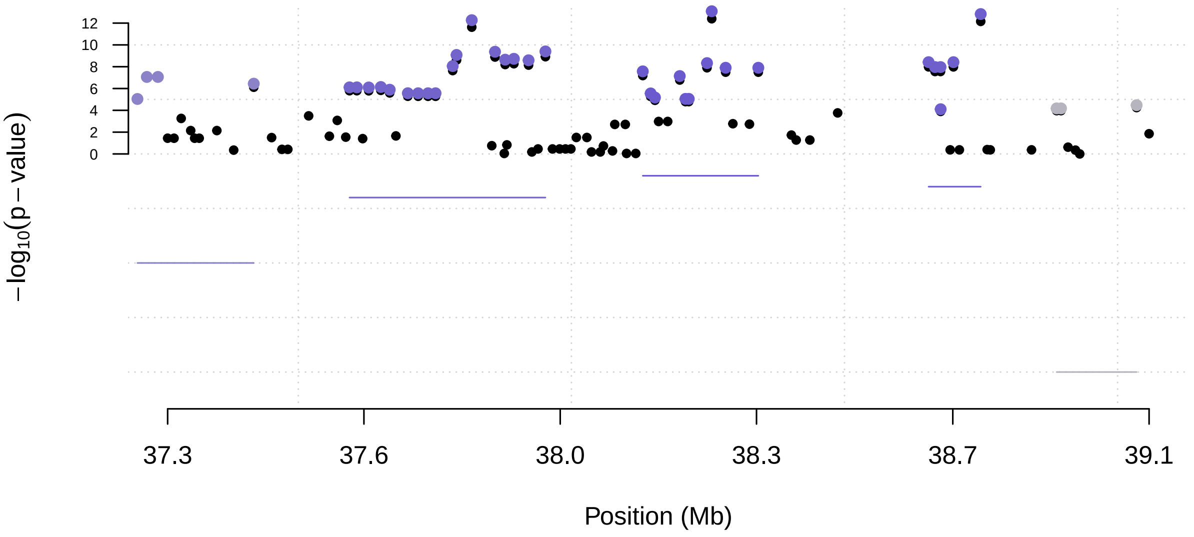

Improved quantile-quantile plots

A quantile-quantile plot (QQ plots) is a graphical way of comparing two probability distributions. In GWAS studies, QQ plots are commonly used to compare computed per marker p-values with the ones expected under the null hypothesis of no association. This comparison is then used to compute genomic-inflation factor λ which is a good measure of the degree of confounding caused by population structure. The **cgmisc** package provides the `plot.qq` function for better visualization and improved interpretation of standard QQ plots. The function plots expected vs. observed distribution of p-values and shows theoretical confidence interval computed using approach outlined in (Casella and Berger 2002). Apart from showing the theoretical confidence interval, it can also perform a number of randomization tests (shuffling the phenotype) to determine empirical confidence interval which, due to LD, is often narrower than the theoretical one. Empirical p-values can be supplied by the user, otherwise a randomisation test on Grammar gamma-transformed residuals is performed as outlined in (Belonogova et al. 2013).

```r
# Perform permutation tests
h2h <- polygenic(formula = ct ~ sex,
                 kinship.matrix = data.qc1.gkin,
                 data = data.qc1,
                 llfun = 'polylik')
result <- c()
h2h.tmp <- h2h
pb <- txtProgressBar(min = 0, max = 10, initial = 0, style = 3)
for (i in 1:10) {
  h2h.tmp$grresidualY <- sample(h2h$grresidualY)
  tmp <- qtscore(h2h.tmp$grresidualY,
                 data = data.qc1,
                 strata = pop,
                 clambda = F)
  result <- rbind(result, sort(-log10(tmp@results$P1df)))
  setTxtProgressBar(pb, i)
}
```

```r
plot.qq(data = data.qc1,
        obs = mm[,'P1df'],
        emp = h2h,
        N = 10,
        plot.emp = T, step = 100)
```

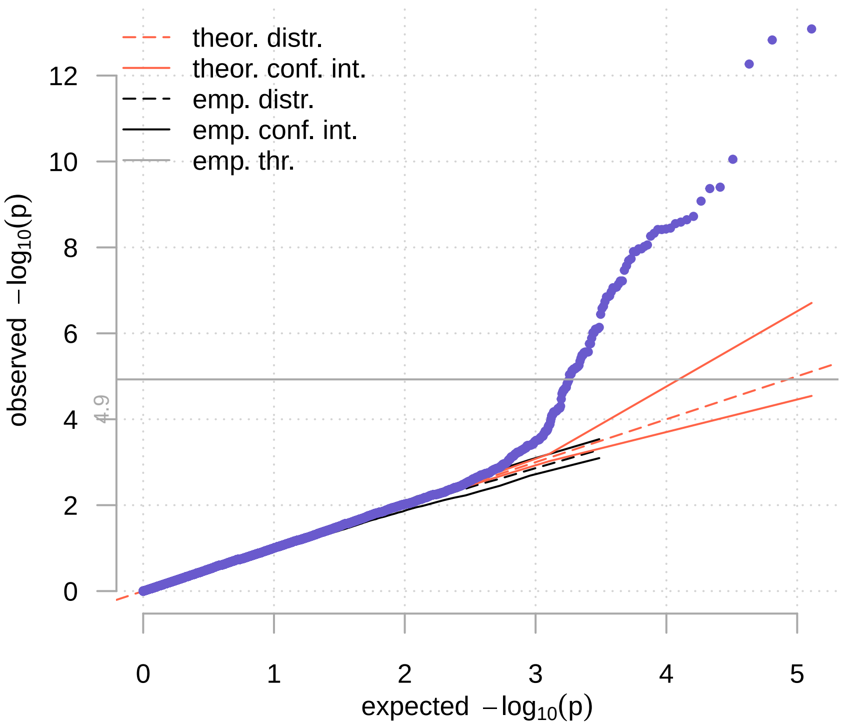

Interacting with the UCSC genome browser

It is often convenient to display p-values from a genome-wide association scan directly in UCSC Genome Browser (Kent et al. 2002) to easily align annotations with the signals of interest. This can be done with `gwaa.to.bed` function that exports coordinates and p-values from `gwaa.data-scan` into a BED file that can easily be used to set a custom path in the UCSC Genome Browser.

Using the `open.region.ucsc` function, it is possible to automatically open an Internet browser window containing UCSC Genome Browser result for a set of predefined genomic coordinates and assembly of interest.

```
open.region.ucsc(chr = 2,
                 coords =  c(37256927, 39256927),
                 assembly = 'canFam3')
```

Examining LD decay

To visualise LD decay on chromosome 28, one can call the `plot.ld.decay` function.

```
plot.LD.decay(data = data.qc1[ ,data.qc1@gtdata@chromosome == 28])
```

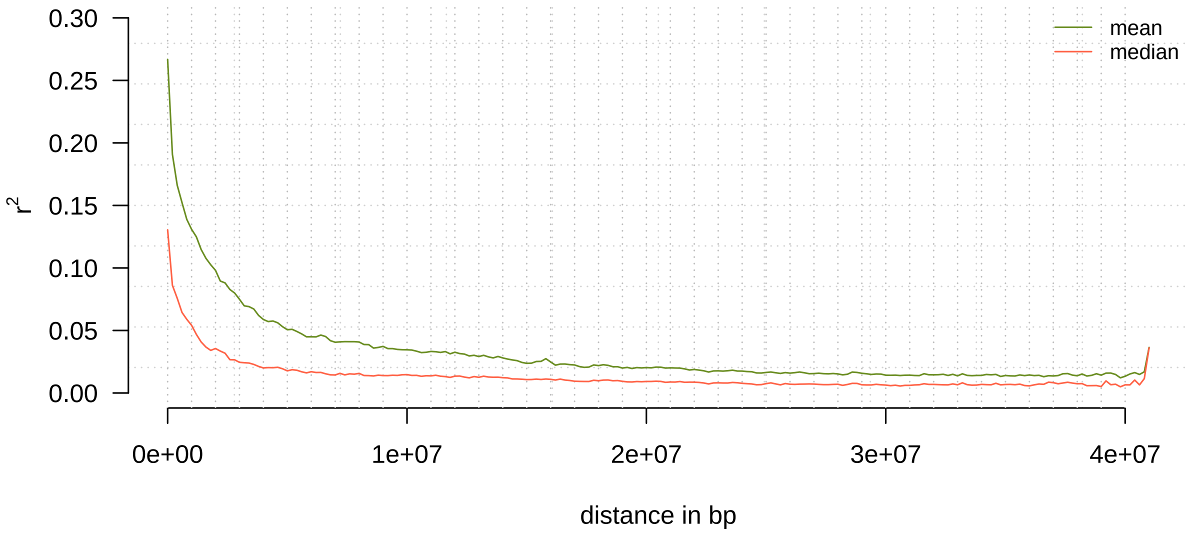

Detecting runs of homozygosity

The `get.overlap.windows` function divides the selected chromosome (or the whole genome) into overlapping chunks of given size and overlap. The function returns a list containing window coordinates along with a logical matrix where each window is represented by a row and the logical value per-marker is set to *true* if the marker is contained within the window and to *false* otherwise. One can specify (in bp) size of a window as well as overlap between windows. If overlap is set to 0, non-overlapping windows will be used.

We will divide chromosome 2 into overlapping windows and then use them to calculate mean heterozygosity and identify runs of homozygosity:

```
my.LW <- get.overlap.windows(data = data.qc1,
                             chr =  2,
                             size = 125e3,
                             overlap = 2500)
```

Computing average heterozygosity using overlapping windows approach

Heterozygosity is evaluated based on allelic frequencies of markers in particular overlapping windows and the basic Hardy-Weinberg theorem equation. The values range from 0 to 1 and correspond to the probability that the given set of loci, represented by the window, is heterozygous.

```
het.windows <- het.overlap.wind(data = data.qc1,
                                LW = my.LW,
                                progress = F)
```

Now, having calculated average heterozygosities we can visualize them with the `plot.overlap` function:

```r
plot.overlap(LW = my.LW, heter.zyg = het.windows)
```

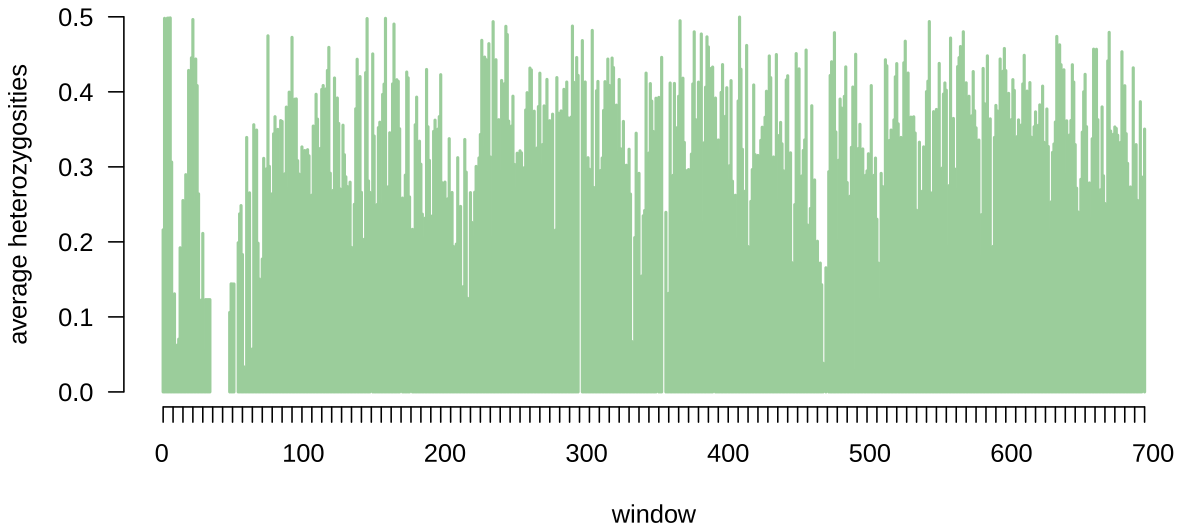

We can use the calculated heterozygosity to detect runs of homozygosity across the selected chromosome or region. We will use the `get.roh` function to check if we have any stretches of reduced heterozygosity on chromosome 2. All windows with the average heterozygosity below a given threshold, here 0.30, be deemed homozygous.

```r
get.roh(data = data.qc1,
        chr = 2,
        LW = my.LW,
        hetero.zyg = het.windows,
        threshold = 0.30,
        strict = TRUE)
```

```
##      window    begin      end length
## [1,]      8  4010290  5360290     87
## [2,]    197 27162790 28022790     54
## [3,]    316 41740290 42477790     52
## [4,]    436 56440290 57667790     77
```

As a result we get a matrix with runs coordinates, length (in windows) and first window that starts a stretch.

Examining allele/genotype effect using phenotype boxplots

For quantitative traits, the `boxplot.snp` function can be used to visually examine allele or genotype effect by plotting phenotype boxplots for the individuals in every genotypic class. The function works for both outbreed (three boxes) and inbreed (two boxes) data.

```
marker <- 'BICF2S2365880'
trait.name <- 'response'
boxplot.snp(data = data.qc1,
            marker = marker,
            trait = trait.name,
            recode = F,
            font = 2)
```

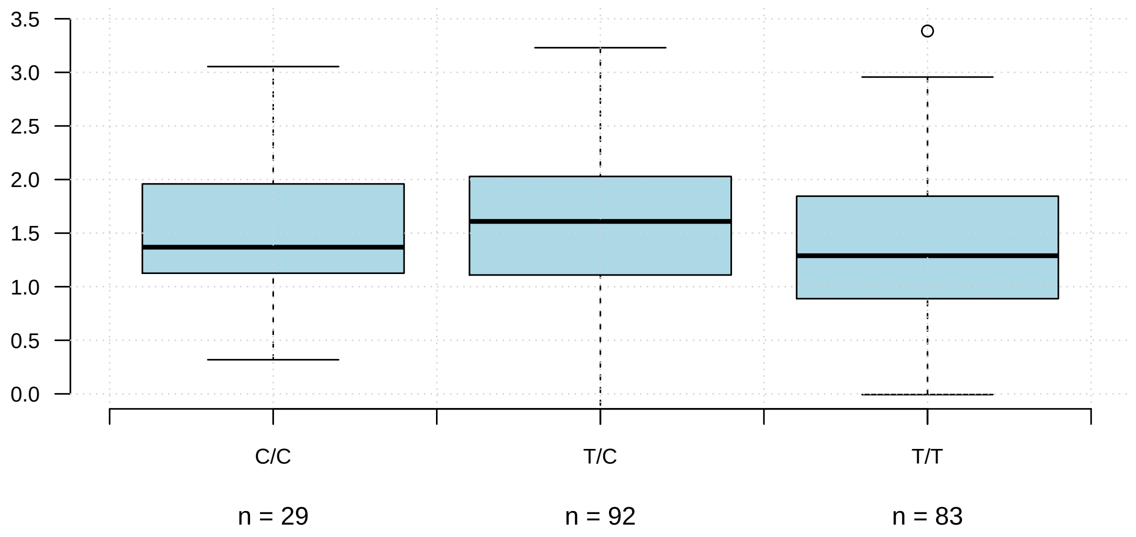

Simple epistasis scan

To gain further understanding of the genetic architecture underlying our trait, we might want to search for potential epistasis between pairs of SNPs. In the function `epistasis.scan`, we implement a simple way of doing this by fitting linear models including two SNPs: $y = \beta_0 + \beta_1 * SNP + \beta_i * SNP_i + \beta_{i,int} * SNP * SNP_i + e$, where y is the phenotype, SNP and SNP_i are the genotypes at the two SNPs, and e is the residual. $i = 1...n$, where n is the number of SNPs in the input to the function. The function takes a SNP, phenotypes, and a `gwaa.data-class object` as input. It then fits linear models between SNP and all markers/SNPs in the `gwaa.data-class` object. To visualize a potential two-SNP interaction, one can use the function `boxplot.snp.twoWay`. This function shows a two-locus genotype-phenotype map by plotting phenotype boxplots for the individuals in every genotypic class, as defined by the two SNPs jointly. This gives nine or four boxes, for outbred and inbred data respectively.

```
es <- epistasis.scan(data = data.qc1[ ,data.qc1@gtdata@chromosome == 2],
                     SNP = 'BICF2S2365880',
                     trait.name = 'response')
```

```
boxplot.snp.twoWay(data.qc1,
                   marker1 = 'BICF2S2365880',
                   marker2 = 'BICF2S2337600',
                   trait = 'response')
```

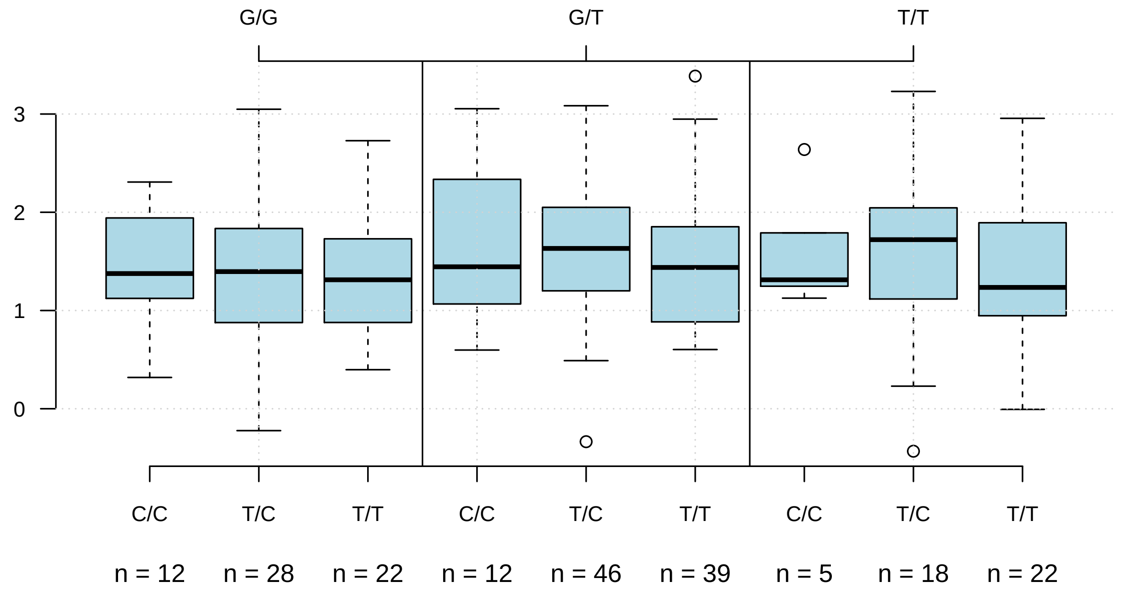

Data conversion functions

Our package provides a number of functions which enable navigating between various data formats such as FASTA or the input format used by the PHASE software.

`gwaa.to.bigrr` The bigRR package implements a computationally-efficient generalized ridge regression (RR) algorithm for fitting models with a large number of parameters (Shen et al. 2013). `gwaa.to.bigrr` function exports gwaa.data-class object to bigRR-compatible input structure.

`gwaa.to.vgwas` In a standard approach to GWAS, associations are detected based on differences in mean phenotypic values across genotype classes. Shen et al. (2012) have proposed an extended approach where the associations are detected based on differences in variances, not means only. They have implemented the approach in the **vGWAS** R package. The `gwaa.to.vgwas` function converts gwaa.data-class object to a format readable by the vGWAS package.

`gwaa.to.phase` enables the user convert a GenABEL `gwaa.data-class` object to the intrnal PHASE input format.

`phase.to.fasta` PHASE is a software for haplotype reconstruction and our `phase.to.fasta` function can be used to parse a part of PHASE output to a customised FASTA format:

```
'>haplo_1_count_176'
'TCGGGCTC'
```

In the above example, the first line contains the name of the haplotype along with its count, the second line provides the haplotype sequence.

`phase.to.haploview` Converts PHASE output format to the Haploview input format. Haploview is a popular software which faciliates haplotype and LD analyses.

`gwaa.to.bed` produces a BED file containing p-values from a pre-defined region of dataset. The file can be used to, e.g. visualize GWAS p-values as the UCSC Genome Browser custom track.

Dog-specific utilities

Current approaches to finding genome-wide associations in diploid organisms often encounter difficulties when analysing non-autosomal parts of the genome, e.g. the sex chromosomes (Young, Kirkness, and Breen 2008). In **cgmisc**, the `chr.x.fix.canfam` function partially addresses this issue by creating an artificial autosome which consists of pseudo-autosomal regions of the X chromosome. This additional autosome can be analysed in a way similar to all other autosomes. Currently, the function is specific to data coming from the domestic dog (*Canis familiaris*, assemblies canFam2 and canFam3.1).

```
chr.x.fix.canfam (data = data.qc1, assembly = 'canFam3')
```

Endogenous retroviral sequences (ERV)

The `get.erv` function returns information about endogenous retroviral sequences (ERV) in the analysed region. At first, we need to obtain a list of ERV sequences in the defined genomic region using the `get.erv` function. In the **cgmisc** package, we provide collection of canine ERVs identified in canFam3.1 assembly but you can also supply any other database.

Let's search for ERVs on chromosome 2

NOTE! In this example, we are using ERV database for the canFam3.1 assembly on canFam2 data which may not be the optimal way. We shall perhaps use the Liftover software to map coordinates between the assemblies.

```
ervs <- get.erv(chr = 'chr2', coords = c(10e6, 40e6))
print(ervs[1:4, ])
```

```
##     id chromosome strand    start      end length score
## 1 2651       chr2      S 10015031 10006437   8594   309
## 2 2653       chr2      S 10128297 10123555   4742   847
## 3 2659       chr2      S 11537301 11525069  12232   319
## 4 2667       chr2      P 13840823 13856123  15300   324
##                                  subgenes
## 1 5LTR PBS MA CA NC Prot IN TM PPT 3LTR
## 2           5LTR PBS MA NC Prot RT 3LTR
## 3        5LTR PBS CA Prot IN TM PPT 3LTR
## 4           5LTR CA Prot RT IN PPT 3LTR
```

Having a list of ERV sequences, we are able to plot them with `plot.erv` function using the same region:

```
plot.erv(chr = 'chr2', coords = c(10e6, 40e6))
```

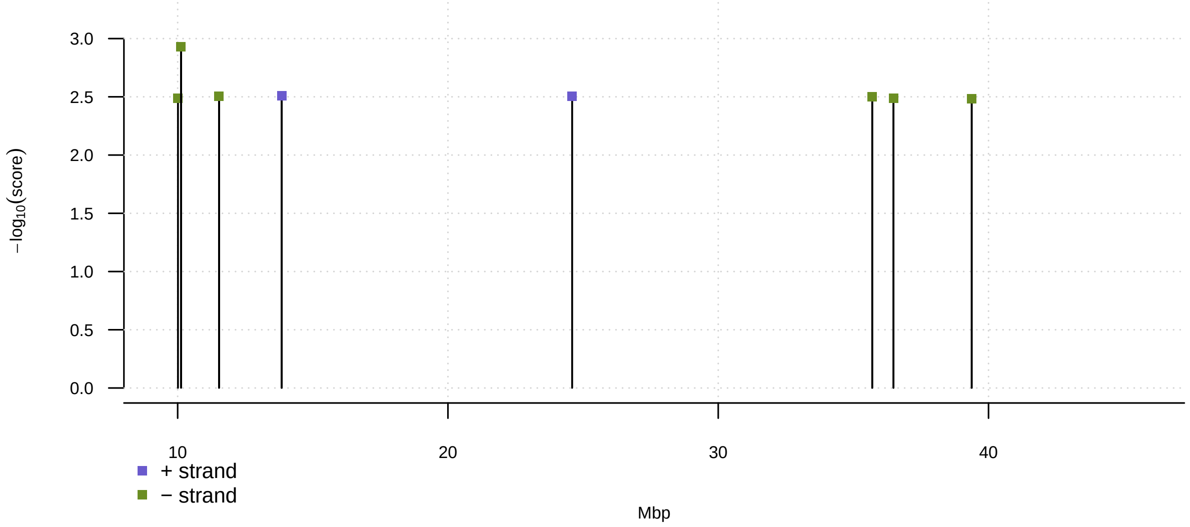

List of function aliases

plot.manhattan.ld: plot.manhattan.LD
plot.ld.decay: plot.LD.decay
phase.to.haploview: phase2haploview, PHASE.to.Haploview, PHASE2Haploview
phase.to.fasta: phase2fasta, PHASE.to.FASTA, PHASE2FASTA
open.region.ucsc: open.region.UCSC
gwaa.to.vgwas : gwaa2vgwas, gwaa.to.vGWAS, gwaa2vGWAS
gwaa.to.phase: gwaa2phase, gwaa.to.PHASE, gwaa2PHASE
gwaa.to.bigrr: gwaa2bigrr, gwaa.to.bigRR, gwaa2bigrr
gwaa.to.bed: gwaa2bed
get.ld.colors: get.LD.colors
plot.fstats: plot.Fst
compute.fstats: compute.Fstats
create.haploview.info: create.Haploview.info

References

Aulchenko, YS, S Ripke, A Isaacs, and CM Van Duijn. 2007. "GenABEL: an R library for genome-wide association analysis." *Bioinformatics* 10 (23): 1294–96.

Belonogova, NM, GR Svishcheva, CM van Duijn, YS Aulchenko, and TI Axenovich. 2013. "Region-based association analysis of human quantitative traits in related individuals." *PloS One* 8 (6): e65395.

Casella, G, and RL Berger. 2002. *Statistical Inference*. Duxbury Advanced Series in Statistics and Decision Sciences. Thomson Learning. http://books.google.se/books?id=0x/_vAAAAMAAJ.

Kent, WJ, CW Sugnet, TS Furey, and KM Roskin. 2002. "The human genome browser at UCSC." *Genome Research*, no. 12: 996–1006.

Shen, X, M Alam, F Fikse, and L Rönnegård. 2013. “A novel generalized ridge regression method for quantitative genetics.” *Genetics* 193 (4): 1255–68.

Shen, X, M Pettersson, L Rönnegård, and Ö Carlborg. 2012. “Inheritance beyond plain heritability: variance-controlling genes in Arabidopsis thaliana.” *PLoS Genetics* 8 (8): e1002839.

Tengvall, K, M Kierczak, K Bergvall, M Olsson, M Frankowiack, FHG Farias, G Pielberg, et al. 2013. “Genome-wide analysis in German shepherd dogs reveals association of a locus on CFA 27 with atopic dermatitis.” *PLoS Genetics* 9 (5): e1003475.

Young, AC, EF Kirkness, and M Breen. 2008. “Tackling the characterization of canine chromosomal breakpoints with an integrated in-situ/in-silico approach: the canine PAR and PAB.” *Chromosome Research : An International Journal on the Molecular, Supramolecular and Evolutionary Aspects of Chromosome Biology* 16 (8): 1193–1202.
